# Meeting Report on the Challenge of Inference from Genome to Phenome

**DOI:** 10.1534/g3.115.019182

**Published:** 2015-10-02

**Authors:** Bevan Emma Huang, Antonio Reverter, Ian Purvis, Scott Chapman

**Affiliations:** * Digital Productivity, Commonwealth Scientific and Industrial Research Organization (CSIRO), Dutton Park QLD 4102, Australia; † Agriculture, Commonwealth Scientific and Industrial Research Organization, St Lucia QLD 4067, Australia; ‡ Agriculture, Commonwealth Scientific and Industrial Research Organization, Armidale NSW 2350, Australia

**Keywords:** G2P, phenomics, transcriptomics, methylation, quantitative genetics

On March 25−27, 2015, in Brisbane, the Commonwealth Scientific and Industrial Research Organization (CSIRO) sponsored a symposium on **The Challenge of Inference from Genome to Phenome**. This conference focused on how the observable characteristics of a living organism (its phenotype) depend on its genotype, its environment, genotype-by-environment interaction, and the way the environment is managed. These questions are fundamental to understanding biological processes, increasing agricultural productivity in plant and animal species, and improving medical care in humans. The challenge of inference has grown exponentially in recent years because of the resolution at which we are now able to measure genotype, environment, management, and phenotype through sequencing, sensing, and other high-throughput technologies. The symposium covered a broad range of topics related to research motivation, measurement, methods, function, and regulation. Keynote speakers were leaders in quantitative genetics, statistics, and functional genomics, with the full program (with links to selected presentations, Supporting Information, File S1) including researchers with experience across plants, animals, and humans. Despite (or perhaps because of) this wide range of experiences, a number of common themes emerged throughout the meeting.

## Get your fundamentals right

Three of the keynote speakers [Rebecca Doerge (Purdue University), Karl Broman (University of Wisconsin-Madison), and Alan Archibald (The Roslin Institute, University of Edinburgh)] stressed the importance, when adopting novel technologies, of first addressing the fundamentals. Whereas Doerge advised to “never marry a technology,” because of their rapidly changing nature, she argued that many of the issues associated with newer technologies such as RNA sequencing (RNAseq) are the same as those faced 10 years ago with the new technologies of that era. She particularly stressed the importance of experimental design, of sample size and replication, of understanding sources of variation, of ensuring quality data to answer questions of interest, and of new computational methods to analyze large datasets. Indeed, these issues were already evident at the dawn of quantitative genetics research early last century. They are still relevant, even if selection and breeding now rely on genomic information in addition to direct measurement of phenotype.

The impact of big and “messy” data also was a central theme for two other speakers. Broman focused on the difficulties of assessing data quality in the realm of very large datasets, and the need for tools to assist researchers to navigate their data. In this realm it can be easy to lose track of fundamentals, such as defining the question of interest, and interpreting the enormous set of results. To combat these challenges, Broman stressed the importance of appropriate and updated training regimes. He also proposed interactive graphic visualizations (R/qtlcharts; Broman 2015) to aid in this endeavor. Jen Taylor (CSIRO) addressed a specific technology producing large datasets—transcriptomics. She raised issues for design and analysis such as understanding the quality of the reference genome for alignment and accounting for the compositional nature of RNAseq samples (considering replication, treatment, organism tissues). Consideration of these issues is vital to produce data that allow interpretation of the function and interconnection of the genome and phenome.

Also addressing the fundamentals of function was Archibald, who through the Functional Annotation of Animal Genomes (FAANG; Andersson *et al.* 2015) is working to provide the kind of genomic resources for farm animals that ENCODE (Encyclopedia of DNA Elements) Consortium (www.encodeproject.org) has for human genetics. Although the prediction of phenotype in domesticated animals has had great success through genomic selection, these fundamental functional genomic resources lag behind those in humans. Hence, this major project aims to produce standardized guidelines and practices, along with a large number of targeted tissue samples from chicken, pig, sheep, and cattle, assays for DNA, histone marks, methylation, and RNAseq to produce functional annotation for a global resource. Complementing this resource, John Williams (University of Adelaide) is producing a resource for functional epigenomics in cattle.

## Phenomics in plants, animals, and humans: What can we learn from each other?

The symposium began with a discussion of the measurement technologies now available across plants, animals, and humans, with researchers sharing their viewpoints on the similarities and differences between these fields. New directions for phenotyping centered on convenience and cost but also on creativity in both devising research questions and ensuring that measurements are taken at appropriate times and environments to answer those questions.

In plants, a primary focus is on dynamic and deep phenotyping. Xavier Sirault (CSIRO), Scientific Director of the High Resolution Plant Phenomics Centre in Canberra, described a range of novel technologies for imaging, dynamic trait capture, and investigation of response to environmental cues such as light and nutrients. Greg Rebetzke (CSIRO) extended this into the practical aspects of delivering ‘omics and physiological traits to breeding programs. The implementation of novel traits requires consideration of trait value (trade-offs, scaling, and location) as well as the impact on selection, and the actual ability to adopt these traits into farming systems.

In animals, traditional phenotyping has focused on convenience and practicality, but new sensors allow more in-depth measurements and the deconstruction of complex phenotypes. Aaron Ingham (CSIRO) demonstrated how sensors on cattle could be used to decompose feed intake into components such as duration, diet, appetite, activity score, and even mitochondrial content. The large flow of data from these sensors requires new methods for handling big data, as well as automated pattern recognition, and statistical analysis to identify the critical time intervals for trait measurement. He and Sonja Dominik (CSIRO) both emphasized the potential for use of these novel traits in selection indices—combining an old approach with new data.

Stuart MacGregor (QIMR Berghofer Medical Research Institute) also highlighted the importance of simultaneously analyzing multiple traits in humans. He described several large datasets with extensive phenotypic and genotypic data (Queensland Twin Registry, 23andMe), illustrating the high level of sharing in medical research. Such large-scale cooperation is less prevalent in other species. As in plant and animal research, dynamic collection of phenotypic data has been facilitated by sensors and wearable devices. The use of real-time, geo-located data from phones and other devices raises many privacy concerns, and in terms of data quality may fall somewhere between “self-reported” and “clinical.” However, compiling and accessing data on behavior and lifestyle more accurately than what could previously only be self-reported, after the fact, will provide a huge source of new information.

## Multifaceted integration increases the scope of investigations

Integration of genome, phenome, environment, and physiological models is moving plant research toward better understanding and prediction of large-scale system dynamics. Keynote speaker Fred van Eeuwijk (Wageningen University) gave a comprehensive overview of genome × environment modeling in plants, from basic models taught in an introductory course for plant breeders, to recent developments incorporating temperature-responsive quantitative trait loci, and characterizing environments through additional variables such as temperature, rainfall, etc. These recent methods exploit “environmental kinships,” variance structures analogous to genetic kinships estimated from markers, to further improve prediction for new environments. Justin Borevitz (Australian National University) described novel resources in *Arabidopsis*, *Brachypodium*, and *Eucalyptus*, both in terms of panels selectively chosen to represent geographic, phenotypic, and genetic diversity, and technologies for altering light intensity, temperature, color and humidity to investigate responses to climate change. These precision growth environments can provide insight into future crop adaptation, although ultimately “the missing heritability is in the field,” and tightly controlled environments can limit the breadth of conclusions that can be drawn.

Mark Blows (University of Queensland) focused on the complex distribution of genetic variance, because characteristics of pleiotropy will have large effects on multivariate response to selection. He showed that pleiotropy is widespread among expression traits and can occur among a large number of traits with disparate function, making its consideration essential in system genetics. Scott Chapman (CSIRO) demonstrated computer simulation models, pulling many of these threads together, by using information collected from remote sensing technology on field traits and climate information, physiological models, and genetic information to assist breeding programs in wheat. Accounting for the interplay of these many sources of information allows evaluation of molecular breeding strategies and crop management for diverse environments.

## Computational advances update old tricks for new data

In addressing the challenge of inference from genome to phenome, statistical and computational developments are critical. Keynote David Balding (University of Melbourne) updated the interpretation of a fundamental genetic quantity, heritability, in the genome-wide sequence era. Although the notion of an exact measure of relatedness underlies much of classical genetics, this no longer makes sense for kinship and heritability estimates, for which pedigree- and identity-by-descent−based measures do not adequately represent reality. However, the goal of measuring the genetic variance explained by using genomic similarity still remains and can be addressed readily by the use of various definitions of single-nucleotide polymorphism−based kinship. The discussion on genetic variance continued with Matt Robinson (University of Queensland), who investigated the between population genetic variance for human height and BMI in large studies across 14 European countries. Toni Reverter (CSIRO) demonstrated an alternate application of genomic relationship matrices constructed from genetic effects across two pedigree-unlinked populations in livestock. His talk further explored the impact of imperfect linkage disequilibrium between marker and QTL on prediction accuracy.

Although some differences exist across domesticated species, it seems that many analyses are moving toward statistical models that combine analysis methods for small numbers of “large-gene effects” with large numbers of “small-gene effects.” Keynote speaker Mike Goddard (University of Melbourne) demonstrated how the BayesR approach (Erbe *et al.* 2012) can simultaneously discover, estimate, and predict such effects by allowing for separate pools of variants with different effect sizes. This would potentially combine the roles of genome-wide association testing with genomic selection. In his keynote speech, Peter Visscher (University of Queensland) agreed with this convergence of methods and demonstrated their application to methylation as another complex trait encompassing both genetic and environmental aspects. He concluded by describing how methylation chips allow estimation of an epigenetic clock that can be correlated with predictions of mortality in humans.

Karin Meyer (University of New England) reminded us of the need for computational carpentry when dealing with large numbers of individuals and multitrait analyses. More powerful computers may make analyses easier but do not obviate the advantages of exploiting the known structure of matrices, using transformations, storing matrices sparsely, and finding equivalent yet numerically simpler models.

## Insights from the keynote panel

One of the final sessions of the conference brought together the keynote speakers to offer perspectives on two general questions. First, the speakers addressed general trajectories in genome to phenome research. There was strong acknowledgment that investments in human genetics are helping to develop tools, whereas resources and investments in agriculture are particularly guiding understanding of genetic architecture, selection, and inheritance. However, there are differences across species and industry in funding—researchers in agriculture are challenged by the lack of drivers to make data sharing more attractive, unlike in medicine, where taxpayer funding drives sharing. In terms of specific technologies, Visscher noted the momentum toward whole-genome sequencing and that “humans are becoming the model organism.” The panelists generally agreed that more phenotyping is always needed, as well as more integration across disciplines, and more training in quantitative skills. Exciting developments are expected with improvements in dynamic phenotyping, “mobile” phenotyping, and characterization of interactions with other organisms (microbiome, soil and water metagenomics, etc.).

Second, the panel addressed what kinds of infrastructure and resources (including people) are required to progress toward solving the inference problem for genome to phenome. Both van Eeuwijk and Doerge raised the need for better overlap in funding and standardization of genotyping, phenotyping, and integration of data. This requires universal standards for data management and naming to reduce the probability of errors. Broman and Visscher focused on the bottleneck of effective researchers, both in terms of the number of scientists working in the area, and the quantitative training environments that foster these researchers. Highlighted skills included the ability to manage data, to create useful tools, and an interest in answering questions using data rather than theory. The ability to transfer such skills across different life science domains was further highlighted; plant and animal breeders are in high demand, even in human research institutes. Indeed, Visscher, who now primarily works in medical research, began his career in animal breeding.

The factors contributing to your choice of species with which to work are numerous and varied, including the impact of your research, the research infrastructure, the level of sharing, of both data and researchers, to name a few reasons. One very clear outcome from the symposium was that there is a general convergence across the species, in terms of questions being asked, technologies, data generation, and analytical tools. As we investigate ever more deeply into these complex interactions between genome, traits, and environment, these resources will be vitally important, because they allow the formation of cross-discipline groups that are better equipped to handle the complex studies required to address system-wide questions.
